# Correlation between nutrition, oral health, and different sarcopenia groups among elderly outpatients of community hospitals: a cross-sectional study of 1505 participants in China

**DOI:** 10.1186/s12877-022-02934-7

**Published:** 2022-04-16

**Authors:** Wenting Cao, Aiyong Zhu, Shufen Chu, Qianqian Zhou, Yinghua Zhou, Xiaoping Qu, Qingrong Tang, Yuxia Zhang

**Affiliations:** 1 School of Nursing, Fudan University, Shanghai, 200032 China; 2 Department of Nursing and Health Management, Shanghai University of Medicine & Health Sciences, Shanghai, 201318 China; 3 Department of Nursing, Zhongshan Hospital, Fudan University, No. 180 Fenglin Road, Xuhui District, Shanghai, 200032 China

**Keywords:** Sarcopenia, Elderly outpatients, NRT status, Oral health, Nutrition

## Abstract

**Background:**

Studies have rarely explored the association between oral health status and different sarcopenia groups (possible sarcopenia, diagnosed sarcopenia, and severe sarcopenia). Moreover, these studies have not reported any definitive conclusions of their relationship. We aimed to characterize the oral health status, prevalence of sarcopenia, and risk factors in different sarcopenia groups of elderly outpatients of community hospitals. Furthermore, we determined the correlation among nutrition, oral health, and different sarcopenia groups.

**Methods:**

Overall, 1505 elderly participants (aged ≥ 65 years) completed the survey. The Mini Nutritional Assessment short-form (MNA-SF) was used to assess the nutrition status of the elderly. Oral health was assessed using the instrument of the oral health assessment index of the elderly (General Oral Health Assessment Index [GOHAI]), and the number of remaining natural teeth (NRT) was counted. Data on muscle mass, muscle strength, and gait speed were collected, and sarcopenia was classified into three groups (possible sarcopenia, diagnosed sarcopenia, and severe sarcopenia) according to the Asian Working Group for Sarcopenia 2019. Multinomial logistic regression multivariate analysis was used to test their relationships.

**Results:**

Eighty-eight (5.8%) participants were identified as having possible sarcopenia; 142 (9.5%), diagnosed sarcopenia; 136 (9.0%), severe sarcopenia; and 1139 (75.7%), no sarcopenia. Of the seven variables, advancing age was typically associated with an increasing prevalence of sarcopenia (odds ratio [OR] = 1.06–1.47, 95% confidence interval [CI] = 1.06–1.47). The results showed that household income (OR = 0.57, 95% CI = 0.33–0.98), education level (OR = 3.32, 95% CI = 1.09–10.07), and chronic diseases (OR = 0.34, 95% CI = 0.19–0.62) were significantly associated with the severe sarcopenia group. Physical activity scores were significantly associated with the diagnosed sarcopenia and severe sarcopenia groups. Participants with < 20 NRT were more likely to have diagnosed sarcopenia (OR = 5.55, 95% CI = 3.80–8.12) or severe sarcopenia (OR = 6.66, 95% CI = 4.13–10.76) than participants with > 20 NRT. The GOHAI score was associated with the diagnosed sarcopenia (OR = 5.55, 95% CI = 3.80–8.12) and severe sarcopenia (OR = 6.66, 95% CI = 4.13–10.78) groups. The MNA-SF score was associated with the different sarcopenia groups.

**Conclusions:**

Assessing early and improving lifestyle with respect to nutrition and oral health may be an effective way to reduce or delay the occurrence of sarcopenia.

## Background

With the aging of elderly individuals, the status of their oral health is not optimal [1]. Some, especially those who have limited economic conditions, may have oral problems such as tooth loss, dental caries, dry mouth, periodontal disease, and cancerous lesions [1]. These oral problems may affect food selection and nutritional intake and may finally lead to frailty, malnutrition, and sarcopenia [2].

Sarcopenia is a syndrome characterized by a decline in skeletal muscle mass together with low muscular strength, physical performance, or both [3]. A previous study demonstrated the relationship between sarcopenia and some adverse health outcomes such as falls, disability, hospitalization, and mortality, which indicates the importance of sarcopenia in health care for the elderly [4].

In recent years, the relationship between sarcopenia and healthy lifestyles of the elderly, such as regular exercise and supplementary nutrition, has attracted much attention. Experts encourage the elderly of any age to improve their physical activity levels and provide safe exercise programs, which are the cheapest and effective ways to counteract sarcopenia [5]. Nutrition such as low protein and energy intake is also linked to sarcopenia [6]. Some experts proposed a concept of oral hypofunction [7], and it is assumed that poor oral health, such as the number of natural teeth, occlusal force, and oral hygiene, would affect food choice and nutrient intake of the elderly, leading to malnutrition and eventually leading to weakness and sarcopenia [8–10]; however, other studies reported no significant associations [2, 11].

According to the latest recommendations of the Asian Working Group for Sarcopenia 2019 (AWGS 2019), sarcopenia was classified into three categories: “possible sarcopenia,” “diagnosed sarcopenia,” and “severe sarcopenia” [12]. Most studies discussed only the correlation between only one or two diagnostic indicators of sarcopenia and oral health status but rarely explored the association between oral health status, nutrition, and different sarcopenia groups. Definitive conclusions of their relationship could not be drawn.

In this study, we aimed to characterize oral health and nutrition, the prevalence of sarcopenia, and risk factors in different sarcopenia groups of elderly outpatients of community hospitals. In addition, we determined the correlation among oral health status, nutrition status, and different sarcopenia groups.

## Methods

### Study design, setting, participants, and sample size

This cross-sectional study was approved by the Medical Ethics Committee of Shanghai University of Medicine and Health Science (2021-bshkt-02–342,524,198,707,240,548), and written informed consent for participation was obtained from all participants. All methods were performed in accordance with the relevant guidelines and regulations. From April 2020 to June 2021, we selected older outpatients of Zhoupu Community Hospital, Jiading Community Hospital, and Nanhui Community Hospital (Shanghai, China). Trained investigators obtained information through health examination (for data on handgrip strength, gait speed, and muscle mass) and questionnaires (for data on basic characteristics, oral health status, and nutrition status).

This was a series study of sarcopenia, and we have reported a part of our results in this manuscript. The sample size was calculated by studying the risk prediction model of sarcopenia among the elderly in the community. According to the multivariate logistic regression, the sample size should be at least 10 times the number of independent variables [13]. There are two dependent variables (non-sarcopenia and sarcopenia) in the study, and we assumed that there were 10 meaningful independent variables. Therefore, the sample size of the case group in this study is about 10 × 10 = 100 cases. Combined with the literature, the incidence of sarcopenia in the elderly in the community is about 10% ~ 20% [12]. Taking 10% as the estimation, the sample size required for the risk model in this study is at least 100 / 10% = 1000 cases. According to logistic regression model requirements, the sample size of the validation model is about two-third of the total sample size. The sample size of the model is about one-third of the total sample size. Then, the total sample size was about 1500.

### Inclusion criteria

- patient’s age ≥ 65 years
- patients who voluntarily participated in the study and signed the informed consent

### Exclusion criteria

- patients with severe cognitive impairment, who cannot understand the content of the questionnaire;
- patients with other serious physical diseases who cannot stand up and walk, such as severe spinal, limb bone, and joint diseases;
- patients with acute diseases of heart, liver, kidney, lung, and other important organs.

### Measures

#### Sarcopenia

Sarcopenia-related parameters include muscle mass, muscle strength, and physical performance. According to the latest criteria of the AWGS 2019 [12], possible sarcopenia is defined by low muscle strength with or without reduced physical performance. Diagnosed sarcopenia is defined by low muscle mass together with low muscle strength or slow gait speed. Severe sarcopenia is defined by low strength, low muscle mass, and low physical performance. Particularly, we obtained relevant data through the following methods, and the cutoff of the sarcopenia parameters was as follows.

#### Muscle mass

Several techniques can be used to assess muscle mass. Considering the cost, availability, and ease of use, we chose bioimpedance analysis (BIA) (InBody 720) to assess appendicular skeletal muscle mass (ASM) [14]. The relative muscle mass index (RSMI) was calculated using the following equation: RSMI (kg/m^2^) = ASM (kg)/height^2^ (m^2^). According to the AWGS 2019 recommendations, the cutoff points for low muscle mass in sarcopenia diagnosis is < 7.0 kg/m^2^ in men and < 5.7 kg/m^2^ in women by BIA [12].

#### Muscle strength

In our study, handgrip strength was used to indicate skeletal muscle strength. The electronic hand dynamometer (CAMRY; China) was used to measure the grip strength of the elderly subjects’ dominant hand. The elderly patient was in the sitting position during the measurement, and the maximum value of three consecutive measurements was chosen. The AWGS 2019 recommends that handgrip strength of < 18 kg in women and that of < 28 kg in men were defined as handgrip weakness [12].

#### Physical performance

General gait speed is the most frequently used test, which is closely related to the occurrence of disability, severe mobility restriction, and mortality [15, 16]. Participants were asked to walk 6 m at a normal pace from a moving start, and they could use walking aids if they were accustomed to it. The walking time was timed using a stopwatch. Low gait speed was defined as gait speed lower than 1 m/s [12].

#### Oral health

An oral health examination was conducted by the investigators. The data of oral health, including the number of remaining natural teeth (NRT) and the oral health assessment index of elderly patients (General Oral Health Assessment Index [GOHAI]). In previous studies, an NRT of ≥ 20 was considered necessary to maintain adequate masticatory function. Therefore, participants were divided into two groups: NRT of ≥ 20 and NRT of < 20 [17, 18]. The GOHAI is an oral health-related QOL instrument that was originally developed in the United States for use among the elderly [19]. Other language versions of the GOHAI, including the Chinese version, have been developed in recent years. The Cronbach’s alpha was 0.81 [20]. The GOHAI contains 12 questions, with three aspects related to physical/functional, psychosocial/psychological, and pain/discomfort. Each question was scored between 1 and 5, and the total score of the 12 questions was considered as the GOHAI score (maximum, 60 and minimum, 12), with a higher score indicating better oral health.

#### Nutrition status

The Mini Nutritional Assessment short-form (MNA-SF) was used to assess the nutrition status of the elderly. MNA-SF is sensitive, specific, and accurate in identifying nutrition risk [21]. The total score on the scale is 14. The higher the score, the better the nutritional status. In addition, 0 ~ 7 points indicate malnutrition, 8 ~ 11 points indicate a risk of malnutrition, and 12 ~ 14 points indicate normal nutritional status.

#### Potential confounders of sarcopenia

The potential confounders used in the study were sex, age, income, education level, monthly household income, alcohol use, cigarette smoking, presence of chronic diseases, and physical activity. Age was used as a continuous variable, and income level was classified into three categories: (1) ≤ 3000 RMB, (2) 3000–6000 RMB, and (3) ≥ 6000 RMB. Education level was classified into three categories: (1) Primary school and below, (2) middle school or senior high school, and (3) university or above. Alcohol use was classified into two categories: (1) No and (2) Yes. Cigarette smoking was classified into two categories: (1) No and (2) Yes. The presence of chronic diseases was classified into two categories: (1) No and (2) Yes. Physical activity was assessed by the instrument of the Chinese version of the Health-Promoting Lifestyle Profile (HPLP-C). The instrument of HPLP-C contains 40 items, of which 8 belong to the dimension of physical activity; 1–4 points for each item. The higher the score, the better the physical activity.

### Statistical analyses

SPSS (version 18.0, Chicago, IL) was used for conducting statistical analyses. Descriptive statistics (mean, standard deviation, and constituent ratio) were used to describe the demographic and clinical characteristics of samples. Chi-square test and Kruskal–Wallis H-test were performed to compare the differences in demographic and clinical characteristics in different sarcopenia groups (non-sarcopenia, possible sarcopenia, diagnosed sarcopenia, and severe sarcopenia). When the differences among several groups were significant, pairwise comparison in multiple independent samples was carried out. Multinominal logistic regression univariate analysis was used to quantify the association between different sarcopenia groups (dependent variable) and confounders (independent variables; sex, age, income level, education level, family income, alcohol use, cigarette smoking, chronic diseases, and physical activity score). Multinominal logistic regression multivariate analysis was used to evaluate the correlation among nutrition, oral health status, and different sarcopenia groups. Odds ratios (OR) and the respective 95% confidence intervals (CI) were calculated.

## Results

### Participants’ characteristics

**Fig. 1:**
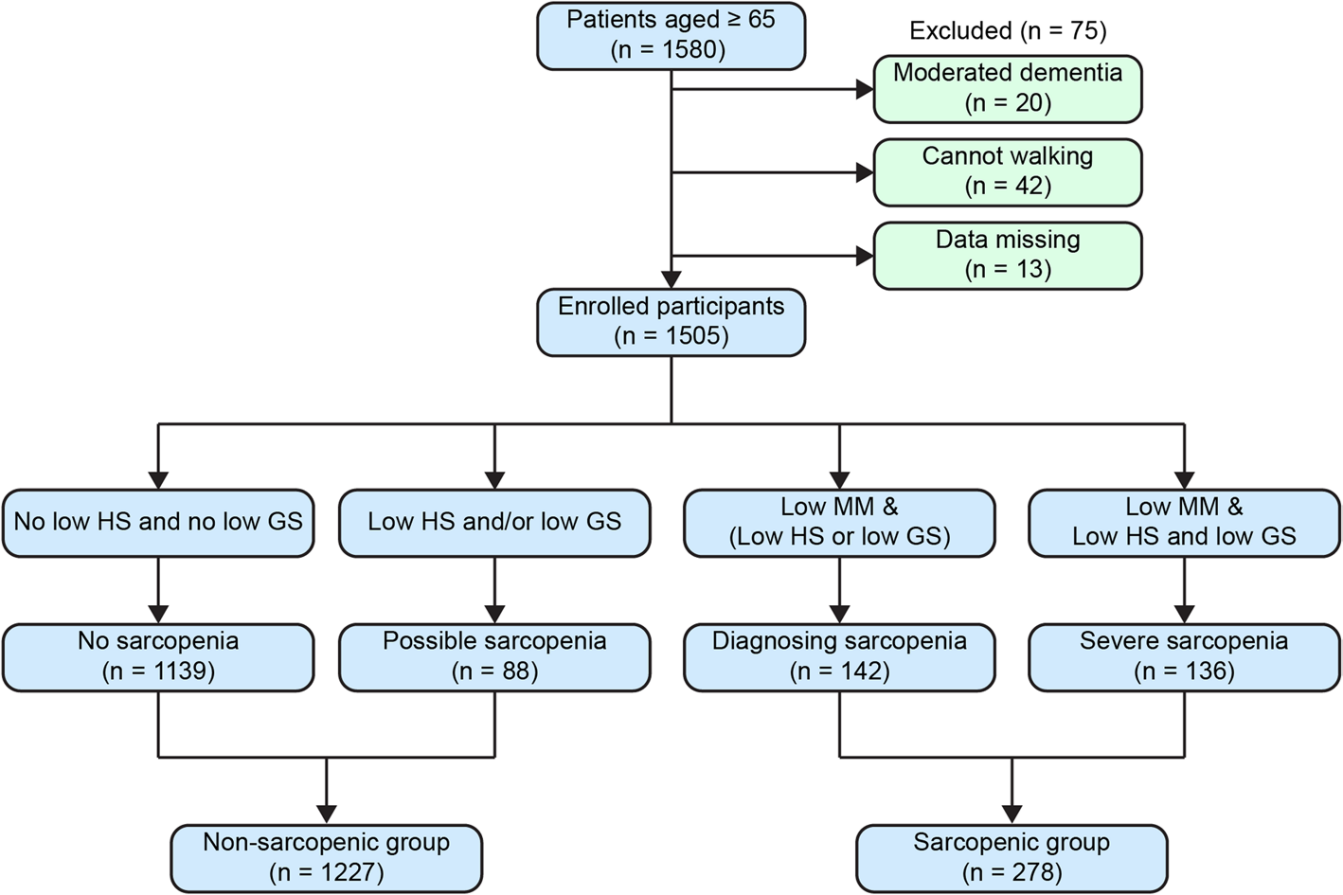
Flowchart showing the study participant selection process and sarcopenia prevalence. GS, gait speed; HS, handgrip strength; MM, muscle mass

A total of 1580 elderly people participated in the survey, and 75 people were excluded. The main reasons for exclusion include dementia, inability to stand, and incomplete data. Notably, 1505 participants who finally completed the questionnaire were included. Figure 1 illustrates a flowchart of the study participant selection process.

According to the AWGS 2019 diagnostic criteria of sarcopenia, 88 (5.8%) participants were identified as having possible sarcopenia; 142 (9.5%), as having diagnosed sarcopenia; 136 (9.0%), as having severe sarcopenia; and 1139 (75.7%), as having non-sarcopenia.

Table 1 shows the sociodemographic and clinical characteristics of the study participants. There were significant differences in age, presence of chronic diseases, and physical activity score among different sarcopenia groups, whereas there were no significant differences in sex, household income, education level, drinking, and smoking. After pairwise comparison of multiple independent samples, we found that the age differences among different sarcopenia groups were significant except between the possible sarcopenia and diagnosed sarcopenia groups. There was a significant difference in the presence of chronic diseases and physical activity scores between the non-sarcopenia and diagnosed sarcopenia groups.

**Table 1:** Sociodemographic and clinical characteristics according to different sarcopenia groups

Characteristics	Non-sarcopenia (*n* = 1139)	Possible sarcopenia (*n* = 88)	Diagnosed sarcopenia (*n* = 142)	Severe sarcopenia (*n* = 136)	*p*-value
Age (years)	74.47 ± 6.23	78.26 ± 6.74	77.02 ± 6.82	85.99 ± 5.83	*p* < 0.001
Sex					*p* = 0.82
Male	487 (57.2%)	34 (61.4%)	64 (54.9%)	58 (57.4%)	
Female	652 (42.8%)	54 (38.6%)	78 (45.1%)	78 (42.6%)	
Household income (RMB)					*p* = 0.10
≤ 3000	315 (27.7%)	28 (31.8%)	33 (23.2%)	44 (32.4%)	
3000–6000	718 (63.0%)	56 (63.6%)	91 (64.1%)	73 (53.7%)	
≥ 6000	106 (9.3%)	4 (4.5%)	18 (12.7%)	19 (14.0%)	
Education level					*p* = 0.06
Primary school or below	152 (13.3%)	18 (20.5%)	10 (7.0%)	16 (11.8%)	
Middle or senior high school	875 (76.8%)	66 (75.0%)	118 (83.1%)	103 (75.7%)	
University level or above	112 (9.8%)	4 (4.5%)	14 (9.9%)	17 (12.5%)	
Alcohol drinking					*p* = 0.36
No	1020 (89.6%)	84 (95.5%)	128 (90.1%)	123 (90.4%)	
Yes	119 (10.4%)	4 (4.5%)	14 (9.9%)	13 (9.6%)	
Cigarette smoking					*p* = 0.61
Non-smoker	909 (79.8%)	73 (83.0%)	112 (78.9%)	114 (83.8%)	
Yes	230 (20.2%)	15 (17.0%)	30 (21.1%)	22 (16.2%)	
Chronic diseases					*p* < 0.001
No	268 (23.5%)	26 (29.5%)	24 (16.9%)	13 (9.6%)	
Yes	871 (76.5%)	62 (70.5%)	118 (83.1%)	123 (90.4%)	
Physical activity score	12.55 ± 3.90	12.51 ± 3.88	10.37 ± 1.72	8.97 ± 1.05	*p* < 0.001
NRT					*p* < 0.001
< 20	165 (14.5%)	13 (14.8%)	71 (50%)	89 (65.4%)	
≥ 20	974 (85.5%)	75 (85.2%)	71 (50%)	47 (34.6%)	
RSMI	7.99 ± 1.62	7.29 ± 1.14	5.75 ± 0.88	5.38 ± 1.00	*p* < 0.001
Handgrip strength	27.59 ± 6.90	21.67 ± 5.60	21.27 ± 4.75	16.37 ± 5.46	*p* < 0.001
Gait speed (m/s)	1.08 ± 0.11	0.97 ± 0.21	0.79 ± 0.19	0.63 ± 0.14	*p* < 0.001
GOHAI	39.26 ± 4.05	39 ± 4.70	37.3 ± 4.47	36.79 ± 4.01	*P* < 0.001
MNA-SF	12.54 ± 0.98	11.11 ± 1.87	10.26 ± 1.76	9.19 ± 1.71	*P* < 0.001

*RSMI* Relative muscle mass index, *NRT* Number of remaining natural teeth, *GOHAI* General Oral Health Assessment Index, *MNA-SF* Mini Nutritional Assessment short-form

Data are presented as mean standard deviation

### Muscle mass

Among women, 156 (18.1%) participants’ muscle mass was less than 5.7 kg/m^2^. Among men, 123 (19.1%) participants’ muscle mass was less than 7.0 kg/m^2^. Table 1 shows the average RSMI of each sarcopenia group, and the difference in RSMI between each group was significant. After pairwise comparison analysis, we found that RSMI differences between the different sarcopenia groups were significant except between diagnosed sarcopenia and severe sarcopenia groups.

### Muscle strength

Among women, 123 (14.3%) participants’ handgrip strength was less than 18 kg. Among men, 110 (17.1%) participants’ handgrip strength was less than 28 kg. Table 1 shows the average handgrip strength in each sarcopenia group, and the difference in muscle strength between the groups was significant. After pairwise comparison analysis, we found that differences in the handgrip strength between different sarcopenia groups were significant except between the “possible sarcopenia group” and diagnosed sarcopenia group.

### Gait speed

Among women, 156 (18.1%) participants’ gait speed was below 1 m/s, and among men, 98 (15.2%) participants’ gait speed was below 1 m/s. The difference in the gait speed between men and women was significant. Table 1 shows the average gait speed of each sarcopenia group, and the difference in gait speed between the groups was significant. After pairwise comparison analysis, we found that differences in the gait speed between the different sarcopenia groups were significant.

#### Nutrition status

Twenty-seven (1.79%) of the participants were assessed as malnutrition (MNA-SF score ≤ 7), 342 (22.66%) were classified as at risk of malnutrition (8≦ MNA-SF score ≦11), and 1137 (75.55%) were classified as normal nutrition (MNA-SF score ≧12. Table 1 shows the MNA-SF score of each sarcopenia group. After pairwise comparison analysis, we found that the differences in the MNA-SF scores between different sarcopenia groups were all significant.

### Oral health status

Among women, 186 (21.6%) participants’ NRT was below 20, and among men, 152 (23.6%) participants’ NRT was below 20. The difference in the NRT between men and women was not significant. Table 1 shows the NRT of each sarcopenia group. There was a significant difference between NRT and different sarcopenia groups. After pairwise comparison analysis, we found that the differences in NRT between the different sarcopenia groups were significant except between the non-sarcopenia and possible sarcopenia groups.

The mean GOHAI score was 38.84 ± 4.21 (range: 21–49). The most common negative impact was the item “teeth or gums are sensitive to cold, hot or sweets.” The lowest negative impact was the item “restriction the communication with others.” Thirty-three (2.2%) patients had a total GOHAI score below 40; 470 (31.2%) people, 40–49; and 1002 (66.6%) people, higher than 50. Data analysis results showed that women’s GHOAI score was significantly better than men’s (women 40.44 ± 3.31 vs. men 36.70 ± 4.45, *P* < 0.001). Table 1 shows the GOHAI score of each sarcopenia group. There was a significant difference between the GOHAI score and different sarcopenia groups. After pairwise comparison analysis, we found that the differences in the GOHAI score between different sarcopenia groups were significant except between the non-sarcopenia group and the possible sarcopenia group and between the diagnosed sarcopenia group and the severe sarcopenia group.

### Risk factors related to different sarcopenia groups

Table 2 shows the risk factors related to different sarcopenia groups. Of the eight variables, advancing age was typically associated with an increasing prevalence of sarcopenia. Physical activity score was significantly associated with the diagnosed sarcopenia group (OR = 0.80, 95% CI = 0.74–0.85) and the severe sarcopenia group (OR = 0.54, 95% CI = 0.47–0.61). Household income (OR = 0.57, 95% CI = 0.33–0.98), education level (OR = 3.32, 95% CI = 1.09–10.07) and presence of chronic diseases (OR = 0.34, 95% CI = 0.19–0.62) were significantly associated with the severe sarcopenia group. However, there were no significant differences between other variables and sarcopenia.

**Table 2:** Risk factors related to different sarcopenia groups

Factors	Possible sarcopenia (*n* = 88)				Diagnosed sarcopenia (*n* = 142)				Severe sarcopenia (*n* = 136)			
	β	OR	95% CI	*p*-value	β	OR	95% CI	*p*-value	β	OR	95% CI	*p*-value
Age^a^	-9.47	1.10	1.06–1.13	*p* < 0.001	-6.68	1.06	1.03–1.09	*p* < 0.001	-29.44	1.40	1.34–1.47	*p* < 0.001
Sex												
Female	-2.66	1.19	0.76–1.85	0.45	-2.03	0.91	0.64–1.29	0.60	-2.13	1.00	0.70–1.44	0.98
Male^b^		1										
Household income												
≤ 3000	0.86	2.36	0.81–6.87	0.12	-0.48	0.62	0.33–1.14	0.12	0.40	0.78	0.44–1.40	0.40
3000–6000	0.73	2.07	0.73–5.82	0.17	-0.29	0.75	0.43–1.29	0.29	0.04	0.57	0.33–0.98	0.04*
≥ 6000^b^		1										
Education level												
Primary school or below	1.20	3.32	1.09–10.07	0.03*	-0.64	0.53	0.23–1.23	0.14	-0.37	0.69	0.34–1.43	0.32
Middle or senior high school	0.75	2.11	0.76–5.91	0.15	0.08	1.08	0.60–1.94	0.80	-0.25	0.78	0.45–1.34	0.37
University level or above^b^		1				1				1		
Drinking												
No		2.45	0.88–6.80	0.09		1.07	0.60–1.91	0.83		1.10	0.60–2.02	0.75
Yes^b^		1				1				1		
Smoking status												
Non-smoker		1.23	0.69–2.19	0.48		0.95	0.62–1.45	0.79		1.31	0.81–2.12	0.27
Yes^b^		1				1				1		
Chronic diseases												
No	26	1.36	0.85–2.20	0.20	24	0.66	0.43–1.05	0.08	13	0.34	0.19–0.62	*P* < 0.001
Yes^b^	62	1			118				123			
Physical activity score^a^	-0.01	0.99	0.94–1.06	0.93	-0.23	0.80	0.74–0.85	*p* < 0.01*	-0.62	0.54	0.47–0.61	*p* < 0.001

*OR* Odds ratio, *CI* Confidence interval

^a^Non-sarcopenia group was the reference

^b^The variable was the reference

^*^The *p*-values were calculated using the chi-square test for categorical variables and using the Kruskal–Wallis H-test for continuous variables

### Correlation among oral health, nutrition, and different sarcopenia groups

Table 3 shows the relationship between sarcopenia and GOHAI score in multinominal logistic regression multivariate analysis. After adjusting for potential confounders, the GOHAI score was associated with the diagnosed sarcopenia group (OR = 5.55, 95% CI = 3.80–8.12) and the severe sarcopenia group (OR = 6.66, 95% CI = 4.13–10.78). However, there was no significant difference in the GOHAI score between the possible sarcopenia and the non-sarcopenia groups.

**Table 3:** Odds ratio estimating effects of the number of natural remaining teeth ^a^ (NRT) on sarcopenia

	Unadjusted^b^ model					Adjusted^c^ model	
	β	OR (95% CI)	P	β	OR (95% CI)		P
Possible sarcopenia^d^	0.023	1.02 (0.56–1.89)	0.94	-0.20	0.82 (0.44–1.53)		0.533
Diagnosed sarcopenia^d^	1.775	5.90 (4.09–8.53)	< 0.001	1.75	5.79 (3.90–8.59)		< 0.001
Severe sarcopenia^d^	2.414	11.18 (7.57–16.51)	< 0.001	1.99	7.32 (4.28–12.52)		< 0.001

*NRT* Natural remaining teeth, *OR* Odds ratio, *CI* Confidence interval

Data are shown as odds ratios (95% confidence interval)

^a^NRT ≥ 20 was the reference

^b^Crude association

^c^Adjusted for age, sex, education level, household income, alcohol drinking, smoking, chronic diseases, and physical activity score by logistic regression

^d^Non-sarcopenia was the reference

Table 4 shows the relationship between sarcopenia and NRT status in multinominal logistic regression multivariate analysis. In the regression model, elderly patients with less than 20 NRT were more likely to have diagnosed sarcopenia or severe sarcopenia than elderly patients with more than 20 NRT. However, there was no significant difference in NRT between the possible sarcopenia and the non-sarcopenia groups.

**Table 4:** Odds ratio estimating the effect of General Oral Health Assessment Index (GOHAI) scores on sarcopenia

		Unadjusted^a^ model		Adjusted^b^ model		
	β	OR (95% CI)	P	Β	OR (95% CI)	P
Possible sarcopenia^c^	-0.017	0.983 (0.931–1.038)	0.540	-0.02	0.98 (0.93–1.03)	0.51
Diagnosed sarcopenia^c^	-0.102	0.903 (0.870–0.938)	< 0.001	-0.11	0.90 (0.86–0.94)	< 0.001
Severe sarcopenia^c^	-0.121	0.886 (0.854–0.920)	< 0.001	-0.06	0.94 (0.89–0.96)	0.04*

*GOHAI* General Oral Health Assessment Index, *OR* Odds ratio, *CI* Confidence interval

Data are shown as odds ratios (95% confidence interval)

^a^Crude association

^b^Adjusted for age, sex, education level, household income, alcohol drinking, smoking, chronic diseases, and physical activity score on logistic regression

^c^Non-sarcopenia was the reference

Table 5 shows the results of correlation among NRT, GOHAI, MNA-SF, and sarcopenia in multinominal logistic regression multivariate analysis. Compared with the non-sarcopenia group, the MNA-SF score had a significant negative impact in the possible sarcopenia group (OR = 0.45, 95% CI = 0.39–0.53), but the results showed that the GOHAI score and NRT had no significant effect in the possible sarcopenia group. In the diagnosed sarcopenia group, the MNA-SF score (OR = 0.35, 95% CI = 0.31–0.41) and NRT (OR = 5.54, 95% CI = 3.57–8.59) had a significant impact in the possible sarcopenia group, but the GOHAI score had no significant effect in the diagnosed sarcopenia group. In the severe sarcopenia group, their correlation was the same as in the diagnosed sarcopenia group.

**Table 5:** Correlation of number of NRT, GOHAI score, and MNA-SF score with sarcopenia

		β	OR (95% CI)	P
Possible sarcopenia^a^	constant	5.28		0.00
	MNA-SF score	-0.79	0.45 (0.39–0.53)	< 0.001
	GOHAI score	0.03	1.03 (0.98–1.09)	0.22
	NRT^b^	0.00	1.00 (0.53–1.88)	1.00
Diagnosed sarcopenia^a^	constant	10.26		0.00
	MNA-SF score	-1.04	0.35 (0.31–0.41)	< 0.001
	GOHAI score	-0.02	0.98 (0.94–1.03)	0.47
	NRT^b^	1.71	5.54 (3.57–8.59)	< 0.001
Severe sarcopenia^a^	constant	13.14		0.00
	MNA-SF score	-1.39	0.25 (0.21–0.30)	< 0.001
	GOHAI score	-0.01	0.99 (0.94–1.04)	0.57
	NRT^b^	2.35	10.50 (6.22–17.72)	< 0.001

*MNA-SF* Mini Nutritional Assessment short-form, *GOHAI* General Oral Health Assessment Index, *NRT* Remaining Natural Teeth, *OR* Odds ratio, *CI* Confidence interval

^a^Non-sarcopenia was the reference

^b^NRT ≥ 20 was the reference

## Discussion

This is the first study to assess the relationship between oral health and different sarcopenia groups according to the latest AWGS 2019 recommendations. It had a large sample size and included extensive data on potential confounders for sarcopenia. Additionally, we expounded on the association between oral health status, nutrition, and different sarcopenia groups.

### Prevalence of sarcopenia

This study demonstrated the prevalence of sarcopenia in different groups. The results showed that the incidence of sarcopenia (including the diagnosed sarcopenia group and the severe sarcopenia group) in elderly patients who were community outpatients was 18.5% (18% in women vs. 19.0% in men). The incidence of sarcopenia was higher than the results of a systematic review conducted in 2020 (11% in men and 9% in women) [22]. A previous study found that the prevalence of sarcopenia varies between 7 and 50%, and it was likely to be affected by age, population type (community-dwelling, nursing home, or hospitals), diagnostic criteria, and muscle mass measurement [23]. In this study, sarcopenia prevalence was age-related, ranging from 23.8% in those aged 70 years to 40% in those aged 80 years in women and from 21.5% in those aged 70 years to 33.3% in those aged 80 years in men. Considering that the participants were recruited from community clinics, most of the elderly patients had chronic diseases (*n* = 1174, 78%), which may have been the reason for the high prevalence of sarcopenia.

### Factors related to sarcopenia

Sarcopenia is a systemic condition, and the related risk factors reported vary greatly. This study found that there were significant differences in age, presence of chronic diseases, and physical activity score, whereas there was no significant difference in sex, education level, household income, cigarette smoking, and alcohol drinking between the different sarcopenia groups. Some studies showed that sarcopenia was more likely to occur in men [24], while other studies reported that sarcopenia was more likely to occur in women [25]. In a sample of Italian community-dwelling older people, duration (in years) of education was inversely associated with the likelihood of being affected with sarcopenia [26]. However, in this study, the two factors had no significant effect on the incidence rate of sarcopenia. Previous studies showed that the presence of chronic conditions and lifestyle factors are associated with sarcopenia. Elderly patients with hypertension, diabetes, and chronic obstructive pulmonary diseases are at risk for sarcopenia [27]. In this study, elderly people with chronic diseases were more likely to have sarcopenia than those without chronic diseases.

### Correlation among oral health, nutrition, and sarcopenia

In this study, the association between NRT and diagnosed sarcopenia and severe sarcopenia was significant; however, there was no significant association between NRT and possible sarcopenia. Some previous studies demonstrated the relationship between NRT and sarcopenia (adjusted OR range: 1.92–2.63) [28, 29]. Tooth loss is a common health problem in the geriatric population, and there is evidence indicating that the number of natural teeth is closely related to chewing ability and dietary intake [30, 31], eventually reducing muscle mass and QOL. Older adults who have a lower number of natural teeth are more likely to avoid some foods such as meat (tough to chew), raw foods, and some dry foods. These foods, which contain protein; calcium; and vitamins A, D, and E; play important roles in the metabolic function of the body and can lead to the loss of muscle mass [32].

Only a few studies have reported the association between the GHOAI score and sarcopenia. In this study, we found that the GOHAI score of women is significantly better than that of men. In the adjusted model, the GOHAI score was significantly associated with the diagnosed sarcopenia group (OR = 0.877, 95% CI = 0.839–0.917) and the severe sarcopenia group (OR = 0.938, 95% CI = 0.889–0.991). However, there was no significant correlation between the GOHAI score and the possible sarcopenia group. In this study, most of the impacts occurred in the domains of physical function and pain and discomfort of the GOHAI, reflecting the functional needs of the elderly participants. A previous study has shown that the GOHAI score was closely related to the QOL of the elderly [33].

Oral health status reflects the cumulative effects of disease and social factors during the lifetime [34]. Poor oral health is often caused by past or current oral bacterial infections, such as periodontal diseases and dental caries. The number of missing teeth and bad oral status can reflect the cumulative inflammatory and infectious status of oral diseases [35]. Previous studies have shown the association of tooth loss with education and income levels in developed and developing countries [36, 37]. A study analyzed the direct and indirect relationships between early socioeconomic status and severe tooth loss in adults [38]. The results showed that emphasizing early parental education level throughout the lifetime may have a long-term impact on severe tooth loss in middle age. Therefore, to alleviate oral inflammation, early interventions such as improving oral health education and regular appointments with the dentist should be carried out in early life stages.

In this study, we found that early assessment and active management of sarcopenia are important for the elderly [39]. This study suggests that healthcare givers, particularly in primary care, should consider an assessment of sarcopenia in the elderly who are exposed to some risk factors (such as low MNA-SF score, dwindling NRT, or low GOHAI score). The treatment of sarcopenia should be mainly patient-centered and combined with activity programs based on resistance and endurance, including or excluding dietary interventions. A systematic review of nutritional interventions for sarcopenia reported the association between micronutrients and weakness [40]. Four of these studies showed that the higher the protein intake, the lower the risk of weakness. Three studies examined the relationship between dietary quality and weakness and showed that dietary quality was negatively correlated with the risk of frailty [40]. Another review reported that appropriate nutritional methods and amino acid supplementation might be considered as a safe treatment option to significantly improve muscle mass, strength, and function in patients with hip fracture and myopenia [41]. Some pharmacological interventions such as testosterone, estrogen, selective modulators of the androgen receptor, ghrelin, anti-cytokines (IL-1, IL-6, and TNF-α), and myostatin inhibitors have been studied to improve muscle quality and function [42].

In this study, we divided sarcopenia into three groups (possible sarcopenia, diagnosed sarcopenia, and severe sarcopenia) according to the AWGS 2019 recommendations. We explored the relationship between oral health status and each sarcopenia group using comprehensive measurements including muscle mass, muscle strength, and physical function. The results provide a better understanding of the association of oral health status with sarcopenia. However, this study has some limitations. First, this was a cross-sectional study of large samples. Although we confirmed that there is a very close correlation between oral health and sarcopenia, the causal relationship needs to be further validated by longitudinal research. Second, in the evaluation of oral health status, we focused only on the number of natural teeth and oral health QOL and did not collect data on relevant oral health such as periodontitis and dentures; therefore, we could not discuss the relationship between these factors and sarcopenia. Finally, this study confirmed the correlation between oral health status and sarcopenia, but how oral health causes sarcopenia needs to be further discussed.

## Conclusions

This study demonstrated that NRT, GOHAI score, and MNA-SF score were significantly associated with diagnosed sarcopenia and severe sarcopenia among elderly outpatients of community hospitals. The main clinical implication of this study was that improving awareness and behaviors regarding oral health care through community outpatient service may be an effective way to reduce or delay the occurrence of sarcopenia. In the future, more longitudinal studies should focus on assessing the association between oral health and sarcopenia and identifying an effective way to reduce the incidence rate of sarcopenia.

## Data Availability

The datasets generated and/or analysed during the current study are not publicly available due to limitations of ethical approval involving the patient data and anonymity but are available from the corresponding author on reasonable request.

## Abbreviations

ASM: Appendicular skeletal muscle
AWGS: Asian Working Group for Sarcopenia
BIA: Bioimpedance analysis
CI: Confidence interval
GOHAI: General Oral Health Assessment Index
NRT: Remaining natural teeth
OR: Odds ratio
QOL: Quality of life
RSMI: Relative muscle mass index
TNF-α: Tumor necrosis factor-α
IL-1: Interleukin-1
IL-6: Interleukin-6
